# Supplementary figures and images for: A conserved maternal-specific repressive domain in Zelda revealed by Cas9-mediated mutagenesis in Drosophila melanogaster
Source: PLoS Genet. 2017 Dec 19;13(12):e1007120. doi: 10.1371/journal.pgen.1007120 (PMC5752043; doi:10.1371/journal.pgen.1007120)

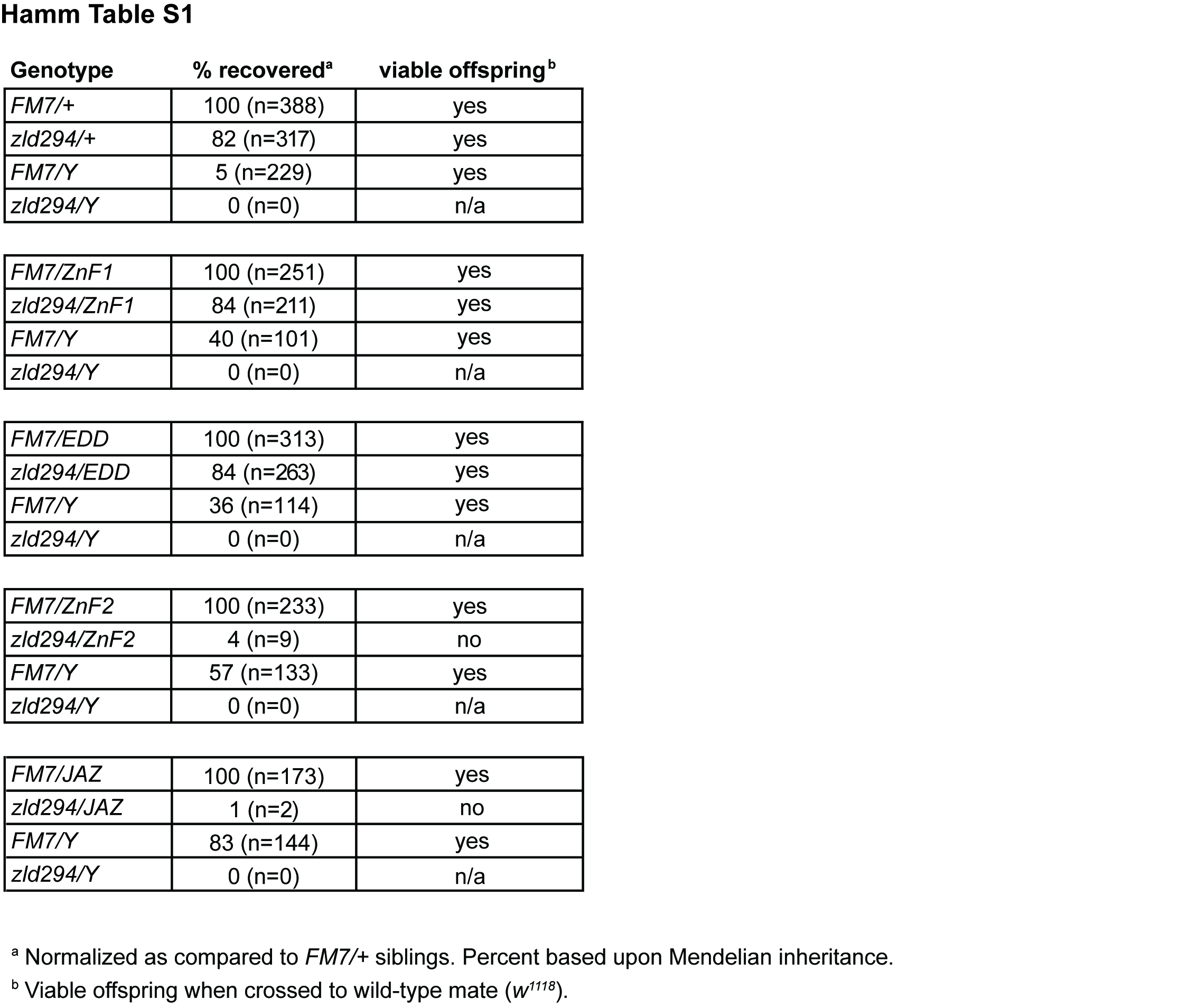

Supplement: S1 Table — Females heterozygous for a deletion allele of zld (zld294) and either the zldZnF1, zldZnF2, zldEDD, or zldJAZ allele were scored for viability and fertility. (TIF) [file pgen.1007120.s001.tif]

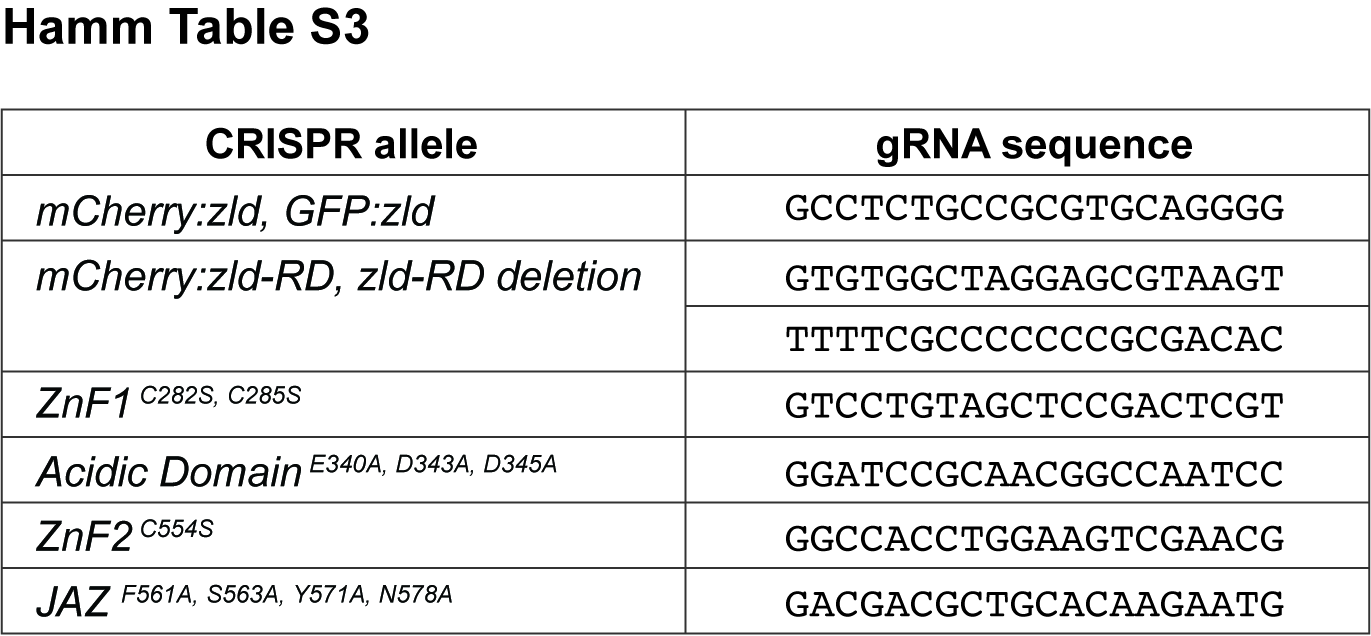

Supplement: S3 Table — (TIF) [file pgen.1007120.s003.tif]

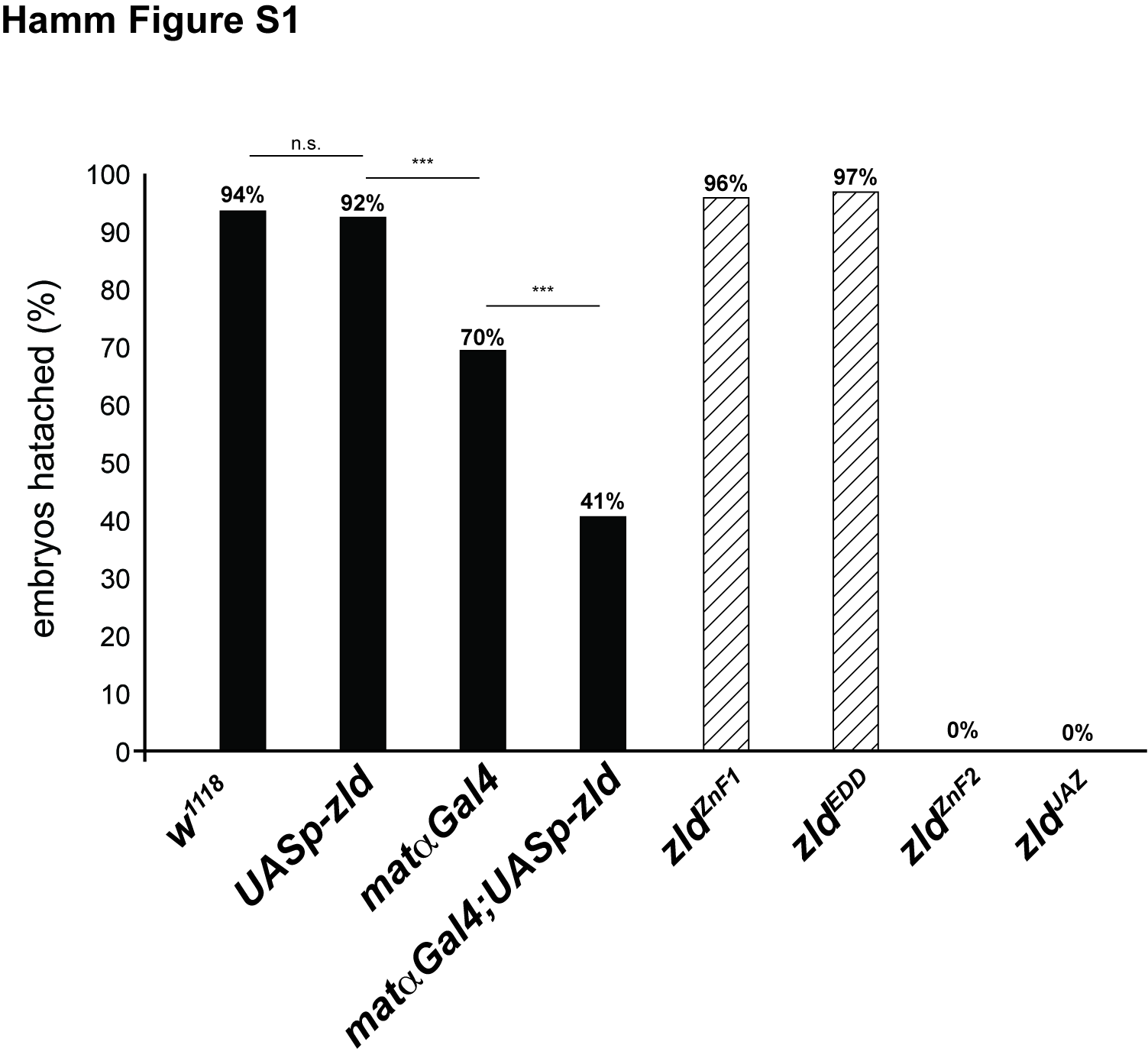

Supplement: S1 Fig — Percent of embryos that hatch from mothers of the phenotypes indicated below. ***, p < 0.0001, calculated by Fisher’s exact test. (TIF) [file pgen.1007120.s004.tif]

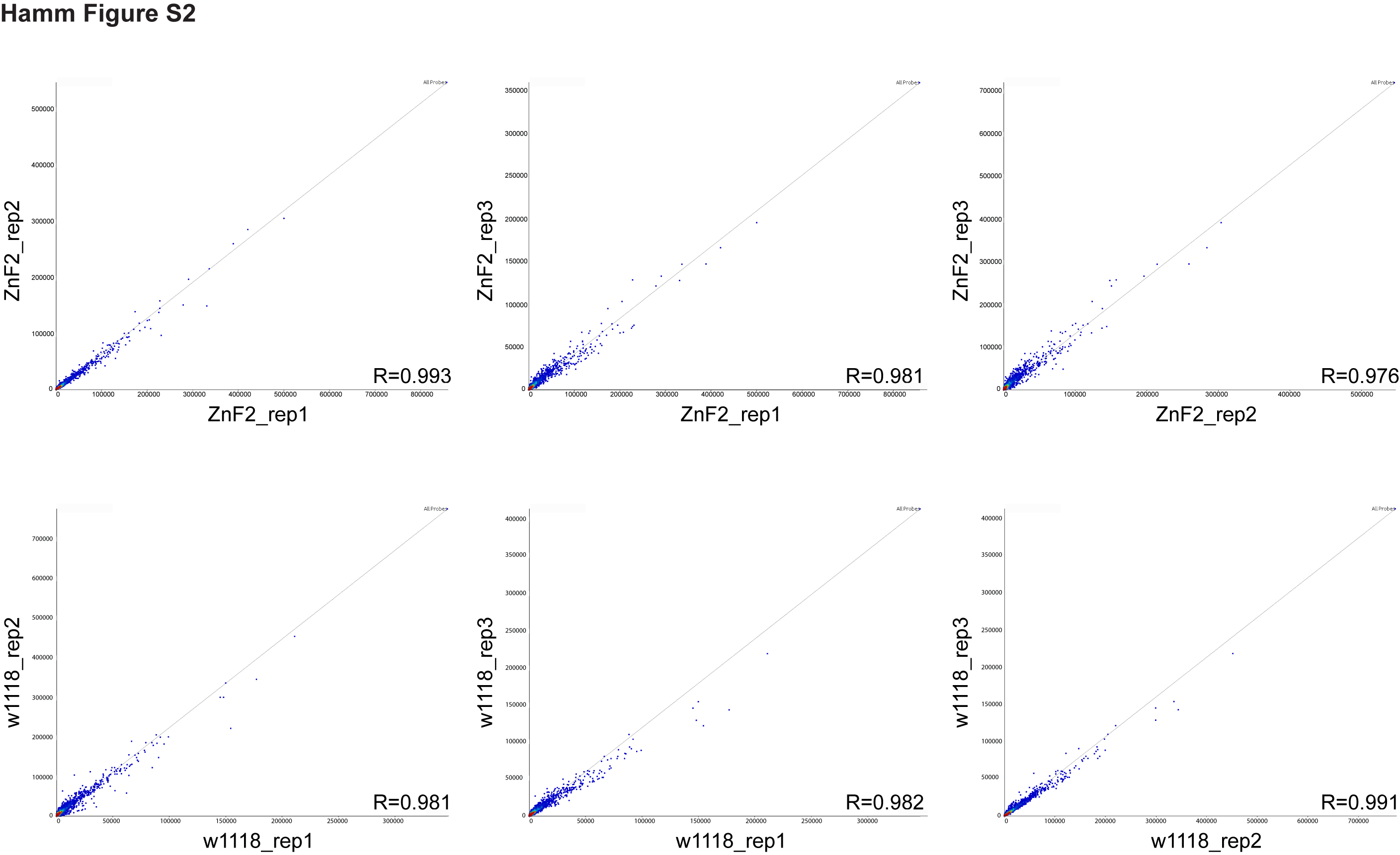

Supplement: S2 Fig — (TIF) [file pgen.1007120.s005.tif]
